# Detection of Time-Varying Structures by Large Deformation Diffeomorphic Metric Mapping to Aid Reading of High-Resolution CT Images of the Lung

**DOI:** 10.1371/journal.pone.0085580

**Published:** 2014-01-13

**Authors:** Ryo Sakamoto, Susumu Mori, Michael I. Miller, Tomohisa Okada, Kaori Togashi

**Affiliations:** 1 Department of Diagnostic Imaging and Nuclear Medicine, Kyoto University Graduate School of Medicine, Kyoto, Japan; 2 Department of Radiology, Kennedy Krieger Institute, Baltimore, Maryland, United States of America; 3 F.M. Kirby Functional Imaging Center, Kennedy Krieger Institute, Baltimore, Maryland, United States of America; 4 Department of Biomedical Engineering, Johns Hopkins University, Baltimore, Maryland, United States of America; 5 Center of Imaging Science, Johns Hopkins University, Baltimore, Maryland, United States of America; Vanderbilt University, United States of America

## Abstract

**Objectives:**

To evaluate the accuracy of advanced non-linear registration of serial lung Computed Tomography (CT) images using Large Deformation Diffeomorphic Metric Mapping (LDDMM).

**Methods:**

Fifteen cases of lung cancer with serial lung CT images (interval: 62.2±26.9 days) were used. After affine transformation, three dimensional, non-linear volume registration was conducted using LDDMM with or without cascading elasticity control. Registration accuracy was evaluated by measuring the displacement of landmarks placed on vessel bifurcations for each lung segment. Subtraction images and Jacobian color maps, calculated from the transformation matrix derived from image warping, were generated, which were used to evaluate time-course changes of the tumors.

**Results:**

The average displacement of landmarks was 0.02±0.16 mm and 0.12±0.60 mm for proximal and distal landmarks after LDDMM transformation with cascading elasticity control, which was significantly smaller than 3.11±2.47 mm and 3.99±3.05 mm, respectively, after affine transformation. Emerged or vanished nodules were visualized on subtraction images, and enlarging or shrinking nodules were displayed on Jacobian maps enabled by highly accurate registration of the nodules using LDDMM. However, some residual misalignments were observed, even with non-linear transformation when substantial changes existed between the image pairs.

**Conclusions:**

LDDMM provides accurate registration of serial lung CT images, and temporal subtraction images with Jacobian maps help radiologists to find changes in pulmonary nodules.

## Introduction

The recent advent of radiological imaging devices has led to an overwhelming amount of anatomical information, which often exceeds the ability of radiologists to inspect within a reasonable reading time. For example, the latest multi-detector row CT (MDCT) can produce images of the entire torso with sub-millimeter high resolution in about 10 seconds, which comprises more than hundreds to thousands of axial slices. For detection and monitoring of a tumor, MDCT is often repeated, which further multiplies the amount of anatomical information. However, this ample information has not been well exploited.

Characterization of the growth or shrinkage of tumor masses, as well as the detection of potentially malignant lesions, are essential parts of a CT-based diagnosis. In clinical practice, tumor size changes are still measured as differences in the longest diameters [1]. Therefore, computer-aided detection (CAD) and quantification of time-dependent anatomical changes are highly desirable. The automated detection of tissue shape change is conceptually straightforward; images from two time points are three-dimensionally registered and a subtraction image is generated [2], [3]. However, the vast majority of our organs are highly deformable, and the registration could be challenging. Among the organs in the human torso areas, the lung is one of the simplest, and therefore, the most researched organs for such automated detection of lesions and their anatomical changes. Nonetheless, precise registration of the lung remains an elusive goal [4], [5].

In order to register two images from the same person with substantial shape changes, highly elastic registration is required. However, such elastic registration could readily be trapped by a local minimum or lead to non-biological severe transformation (e.g., negative Jacobian). For example, the lung has a certain biological topology with two or three lobes in the bilateral thorax with multi-branching bronchi, although their connections are not fully visualized due to the limits of image resolution. Local severe transformation can readily violate this biological topology, which would lead to disconnection or connection of the nearby bronchi. To avoid this type of severe local deformation, a low-dimensional, non-linear transformation, such as polynomial functions, is often used, which would limit the quality of registration.

In this study, we adopted a state-of-the-art diffeomorphic registration tool, called Large Deformation Diffeomorphic Metric Mapping (LDDMM) [6], [7]. This algorithm is specifically designed to cope with a large amount of deformation while retaining the topology of the object; the connected structures remain connected and disconnected structures remain disconnected, even with severe local transformation. LDDMM implementation is featured by a cascading elasticity control, in which the elasticity of the transformation is gradually increased to further enhance the accuracy for cases with severe deformation [8]. This method will enable highly accurate registration and serve as an objective measurement of time-course CT changes.

We applied this new non-linear registration method to serial CT images of lung cancer, and its clinical value, as well as registration accuracy, were evaluated.

## Materials and Methods

### Ethics Statement

This study was approved by Institutional Review Board and Ethics Committee of Kyoto University School of Medicine, and informed consent was waived due to the retrospective nature of this study.

### Patients

Ten primary lung cancer patients and five metastatic lung tumor patients (mean ± S.D.: 65.2±10.3 years old) with two time points of 60.5±26.9 day intervals were recruited from an existing clinical CT database from December 2010 to May 2011. Patient characteristics and diagnoses are listed in Table 1.

**Table 1:** Patient characteristics.

Patients	Sex	Age	Interval between scans (days)	Pathology
1	F	68	35	NSCLC
2	F	61	47	NSCLC
3	F	53	49	NSCLC
4	F	68	36	NSCLC
5	M	66	25	NSCLC
6	M	51	60	NSCLC
7	M	68	44	NSCLC
8	M	72	91	NSCLC
9	M	59	28	SCLC
10	M	67	42	SCLC
11	M	75	86	ascending colon cancer
12	F	44	90	rectal cancer
13	F	72	84	sigmoid colon cancer
14	F	69	106	pancreas cancer
15	M	85	84	gastric cancer
Average ± S.D.		65.2±10.3	60.5±26.9	

NSCLC: Non-small cell lung carcinoma, SCLC: small cell lung carcinoma.

### CT Scans

All examinations were performed with the same 64-slice multi-detector CT scanner (Aquilion 64; Toshiba Medical Systems, Otawara, Japan). Images were acquired with a 1 mm slice thickness, a 0.5 sec rotation time, a beam pitch of 0.83, and 120 kVp of x-ray tube voltage. Automatic exposure control was used for dose reduction. CT scans were conducted after intravenous injection of 2 ml/kg of nonionic contrast medium (300 mg of iodine per milliliter: Iomeprol, Eisai, Tokyo, Japan; Iopamidol, Nihon Schering, Osaka, Japan) at a rate of 2.5 ml/sec in 10 patients. Scan areas were from chest to pelvis in eight patients, and from chest to upper abdomen in seven patients. All scans were reconstructed with a field of view of 350 mm in a 512×512 matrix (0.685×0.685 mm in-plane resolution), with a 1.0 mm slice thickness using a soft tissue kernel (FC13 of the Toshiba CT) to maintain a high signal-to-noise ratio (SNR).

### Image Preprocessing

To detect temporal changes in lung lesions, axial slices that covered the entire lung area of each CT scan were selected, which typically had 150–180 axial slices of 1.0 mm thickness. The images were resampled to 1.0 mm isotropic resolution with the in-plane matrix about 250–350×200, depending on subject’s body size. After proper intensity windowing that clearly revealed the lung structures (window level; −600, window width; 600), the images from the second time point were linearly registered to the first point using 12-mode affine transformation. The linearly registered images were further transformed for more precise image matching using LDDMM. LDDMM registration was conducted according to a previous publication [9].

All image processing for LDDMM was conducted with DiffeoMap (L. Xin, H. Jiang, M. I. Miller, and S. Mori, Johns Hopkins University, www.mristudio.org), which serves as an interface for a Cloud-type computation performed at the Center for Imaging Science, Johns Hopkins University. In this interface, the parameters and were set before LDDMM processing, which determine the elasticity of the transformation (for more details, please see Text S1 in Supporting Information). In brief, the high ratio leads to less elastic transformation, similar to the linear normalization. As the ratio decreases, the transformation is more localized. The level of required elasticity for image registration between the two time-points varies from spatially coarse to highly localized deformation. To deal with clinical cases with a wide range of deformation states, we performed cascading processing, in which three consecutive transformations were performed with ratios of 0.01, 0.005, and 0.002, gradually increasing the elasticity. A cluster computer with 32 CPU and 128 GB of memory was used for LDDMM computation. The computation time varied, depending on the size of the data. For a matrix of 256×192×180 datasets, the non-cascading (single) LDDMM takes approximately one hour, while the cascading LDDMM takes about three times longer.

The performance of LDDMM was compared with B-spline registration, which is commonly used as a non-linear registration method. B-spline registration was applied after affine transformation using a freely available software package, elastix (http://elastix.isi.uu.nl/) [10], with suitable parameters for 3D CT lung images [11]. The cost function was based on mutual information and the registration algorithm was optimized by the stochastic gradient descent methods [12], embedded in a multi-resolution scheme [13], [14]. A grid-size of eight voxels was used in each dimension at the finest resolution level.

### Lesion Size Change and Automated Volume Measurement

LDDMM registration yields a Jacobian determinant map and a transformation matrix. A Jacobian determinant map shows shrinkage or expansion of a lesion as a pixel value smaller or larger than 1, respectively [15]. A transformation matrix can be used to transfer regions-of-interest (ROI) of a tumor at the first time point automatically to the second one, once an ROI is defined manually or automatically using a segmentation tool, which provides a binary mask image of the tumor. The transformation matrix is inversely applied to the first binary mask, which automatically generates an ROI of the nodule at the second time point. For the initial nodule delineation, ROIEditor (X. Li, H. Jiang, and S. Mori, Johns Hopkins University, www.mristudio.org) and its region-growing tool were used.

Temporal subtraction images of the first time point from the second time point were produced at each registration stage (affine, LDDMM with single and that with cascading ). Color-coded Jacobian, which means the determinant of the transformation matrix, was also created, with green for Jacobian <1, red for Jacobian >1, and yellow for Jacobian = 1.

### Accuracy Measurements

**Figure 1:**
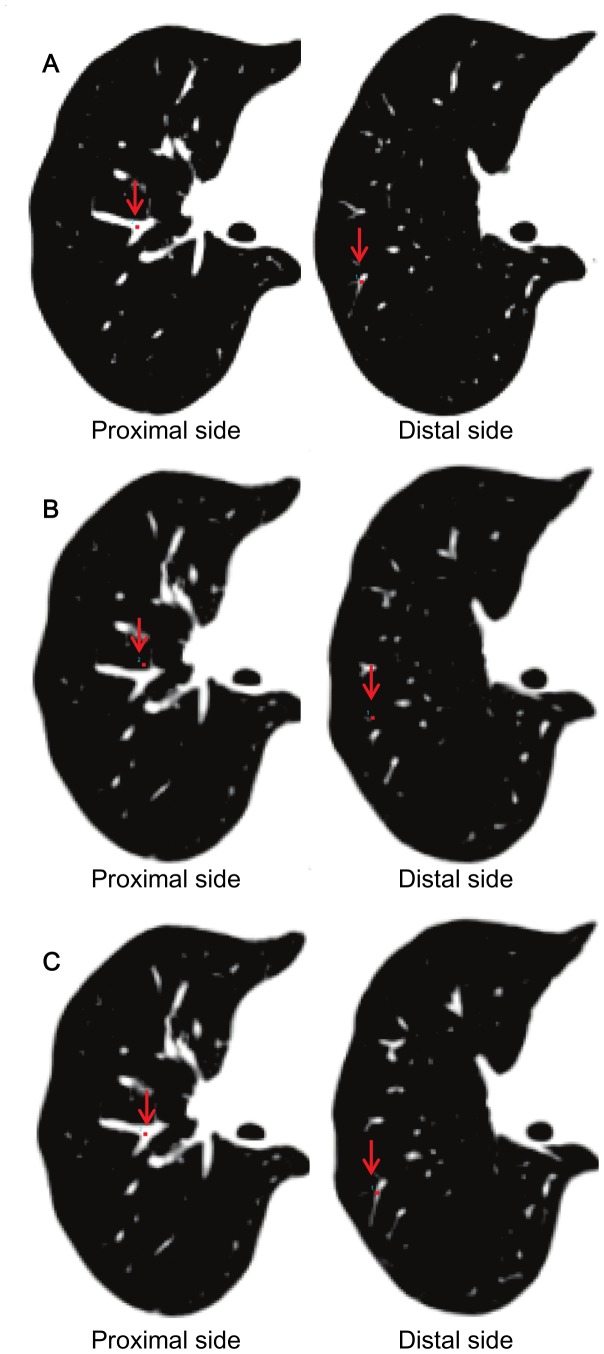
The procedure for the landmark-based registration accuracy measurements by DiffeoMap. A: The 1st time point image with landmarks placed on the distal and proximal bifurcations of vessels (red arrows). B: The landmark coordinates placed on the first time point image were transferred to the linearly registered second time point image, revealing the degree of misalignment. C: Landmarks were manually relocated to the corresponding bifurcations in the 2nd time point images, and the distances of misalignment were measured.

To measure the registration accuracy of the transformation, landmark-based measurements of displacement [3], [16], [17] were calculated using the landmark function of DiffeoMap. As shown in Fig. 1A, landmarks were chosen at the proximal and distal bifurcations of vessels in each lung segment (right lung: 20 landmarks, left lung: 18 landmarks; four landmarks were placed in the left S^1+2^) of the CT image at the first time point. The landmarks were then transferred to the second time point after affine and LDDMM transformation (Fig. 1B) in the same coordinate system. If there was any mismatching between the transferred landmarks and the actual locations of vessel bifurcations (Fig. 1B), they were manually corrected (Fig. 1C). Based on the amount of translation required for remapping of the landmarks, registration accuracy was measured. The relocation of the landmarks was performed three-dimensionally. Bifurcations of the vessels provided unique anatomical landmarks that could be unequivocally defined in the 3D space.

### Statistical Analysis

One-way analysis of variance (ANOVA), followed by post hoc comparisons, were conducted to evaluate differences in average displacements of the landmarks for each proximal and distal side. A p-value of less than 0.05 was considered a statistically significant difference. Statistical analyses were performed using commercially available software (MedCalc, version 12.7.20; MedCalc Software, Mariakerke, Belgium).

## Results

### Registration Accuracy and Nodule Detection

Linear registration often leads to gross misalignment of structures (Fig. 1). The average displacement was 3.11±2.47 mm (mean ± S.D.) and 3.99±3.05 mm, respectively, for proximal and distal landmarks on the affine-transformed second time point images, which decreased to 0.15±0.46 mm and 0.62±1.27 mm, respectively, after single LDDMM transformation, and 0.02±0.16 mm and 0.12±0.60 mm, respectively, after cascading LDDMM transformation. The landmark displacement after B-spline registration was 0.19±0.45 mm and 0.33±0.64 mm, respectively (Table 2). Almost none of the landmarks required repositioning after cascading LDDMM transformation. There were significant differences between every pair of the four types of transformation (p<0.05, post hoc test).

**Table 2:** Registration accuracy.

Transformation type		Linear (affine)	LDDMM with single α	LDDMM with cascading α	B-spline
Landmark displacement (mm)	Proximal	3.11±2.47	0.15±0.46	0.02±0.16	0.19±0.45
	Distal	3.99±3.05	0.62±1.27	0.12±0.60	0.33±0.64
	Average ± S.D.	3.55±2.81*	0.38±0.98*	0.07±0.44*	0.26±0.56*

Displacement of corresponding landmarks on the first time point images and the second time point images was measured as registration accuracy. All pair-wise comparisons showed a statistically significant difference (*p<0.05, post hoc test). Displacements of landmarks were almost zero in cascading *α* LDDMM.

**Figure 2:**
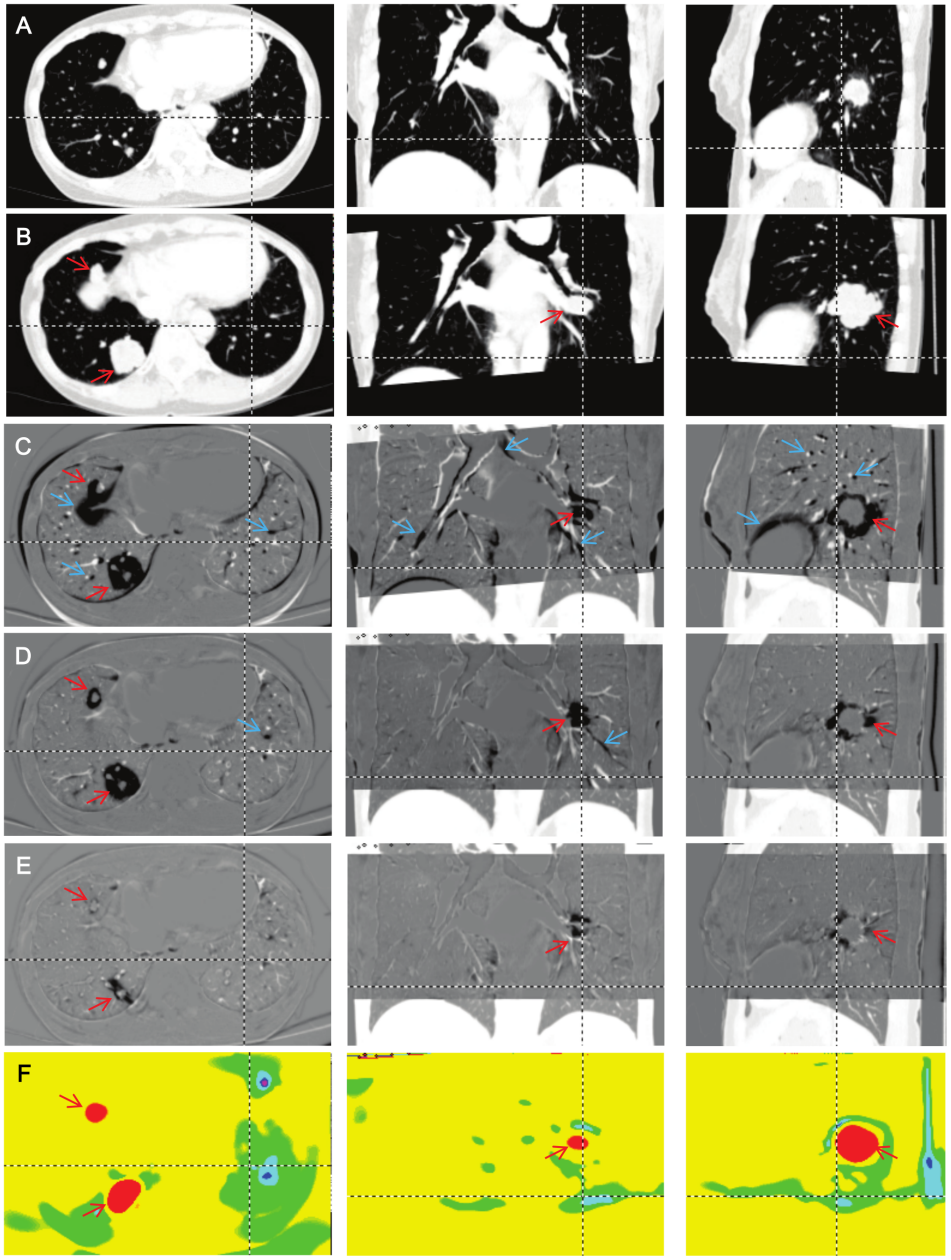
Comparison of three registration results. A: The original first time point image. B: Linearly registered second time point image. Note that only the region of interest was normalized in the second time point. C: A subtracted image between the first time point and the linearly registered second time point. D: A subtracted image with the first iteration of LDDMM (single *α* LDDMM) (*α*/*γ* = 0.01). E: A subtracted image with the cascading *α* LDDMM (*α*/*γ* = 0.01−0.005−0.002). F: A Jacobian map calculated from the transformation matrix of the cascading *α* LDDMM. Red and blue arrows indicate locations of growing tumors (red) and misalignment artifacts (blue). The dotted lines show reciprocal positions.

The linear transformation delivered relatively good registration for the overall anatomy (Fig. 2A, 2B), but the subtraction image revealed numerous mismatching of fine lung structures (Fig. 2C). LDDMM registration with low elasticity with single (Fig. 2D) and high elasticity with cascading (Fig. 2E) demonstrates gradual improvement. The first iteration of LDDMM with less elasticity can drastically improve the registration accuracy, removing most of the misalignment artifacts. While the registration is still not perfect, the decrease in misalignment artifacts would impose less of a burden on readers, and effectively lead their attention to growing nodules. The cascading elasticity control further reduced the remaining misalignment, and subtraction images became less useful (Fig. 2E). In this case, the information about anatomical differences is stored in the transformation matrix, which can be visualized as a Jacobian map (Fig. 2F). We can effectively detect the growing or shrinking nodules.

**Figure 3:**
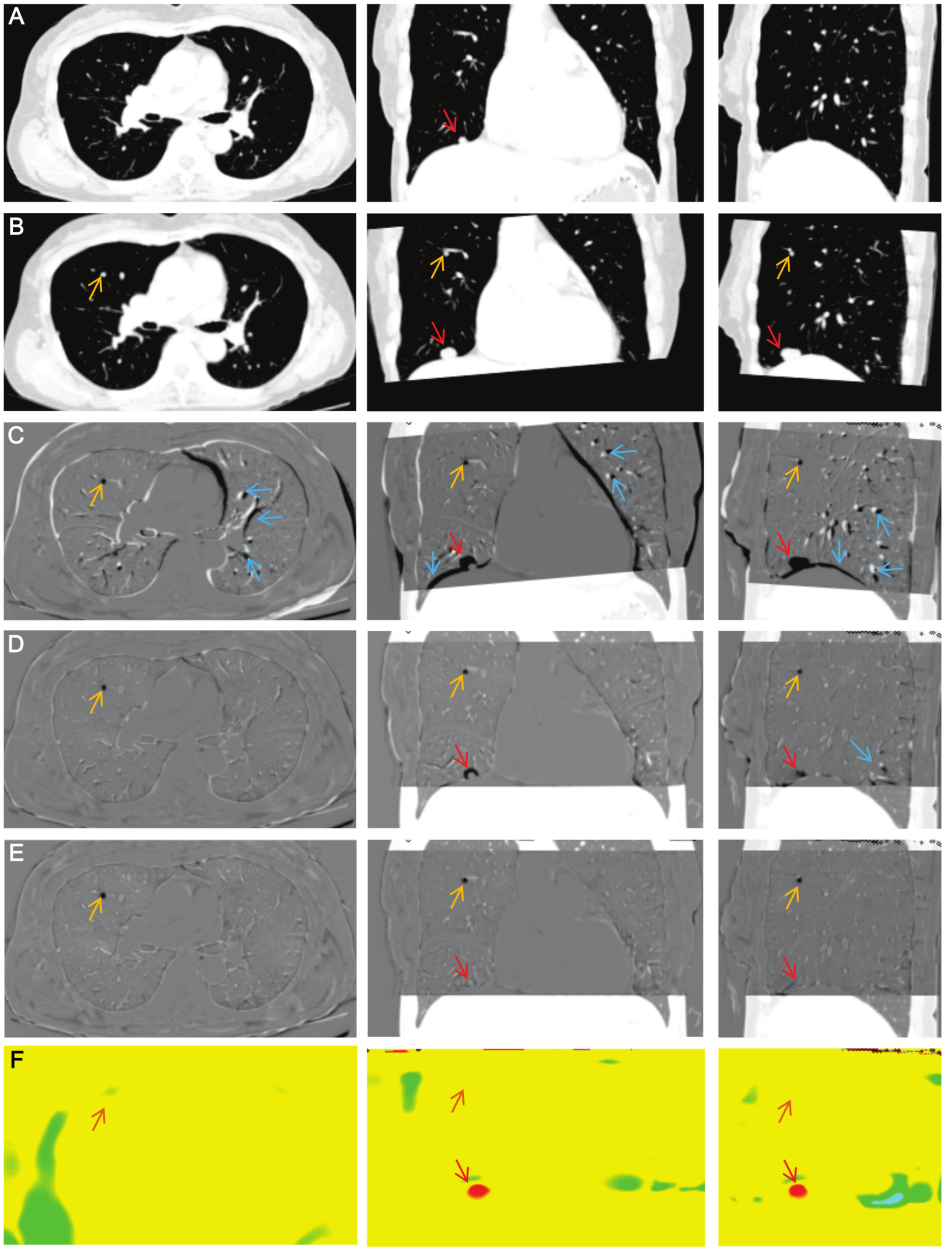
Comparison of growing and emerging tumor. A: The original first time point image. B: The linearly registered second time point image. C: A subtracted image between the first time point and the linearly registered second time point. D: A subtracted image with the first iteration of single *α* LDDMM (*α*/*γ* = 0.01). E: A subtracted image with the cascading *α* LDDMM (*α*/*γ* = 0.01−0.005−0.002). F: A Jacobian map calculated from the transformation matrix of the cascading *α* LDDMM. Orange, red, and blue arrows indicate locations of emerging tumor (orange), growing tumors (red), and misalignment artifacts (blue). Note that almost the entire misalignment is removed by LDDMM, clearly indicating the small nodule that appeared in the second image (orange arrow). This new nodule was not detected by the Jacobian map, which is a metric of growth.

**Figure 4:**
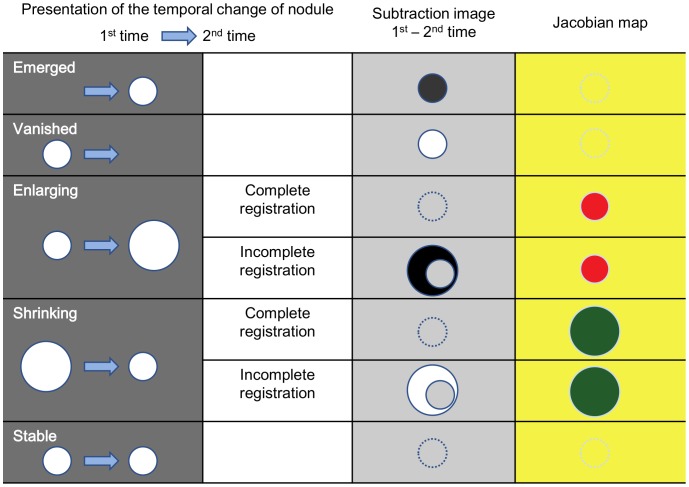
Temporal change in nodules and results of transformation in serial CT scans, with visual presentation in a subtraction image and a Jacobian map. The subtraction image shows gray for pixel value = 0, black for negative, and white for positive value. The color-coded Jacobian map shows green for Jacobian <1, red for Jacobian >1, and yellow for Jacobian = 1.

LDDMM works differently on existing nodules and newly appearing or vanishing nodules. Fig. 3 shows a newly emerged nodule (orange arrow) and a growing nodule (red arrow) detected in one patient. Similar to Fig. 2, there are many misalignment artifacts in the linearly registered images (Fig. 3C), which largely disappear after the first iteration with single *α* (Fig. 3D) and almost completely using the LDDMM with cascading *α* (Fig. 3E). As indicated in Fig. 2, the growing nodule also disappears from the subtraction image (Fig. 3E) and appears as local expansion in the Jacobian map (Fig. 3F). However, the new nodule remains in the subtraction image and is not depicted in the Jacobian map, because it cannot be solved mathematically as local growth. Different patterns of anatomical changes and potential consequences in the subtraction images and Jacobian maps are summarized in Fig. 4. It should be noted that the detection of small metastases could be extremely difficult without a subtraction image or with linearly registered images with registration errors.

### Volumetric Analysis Using the Transformation Matrix

**Figure 5:**
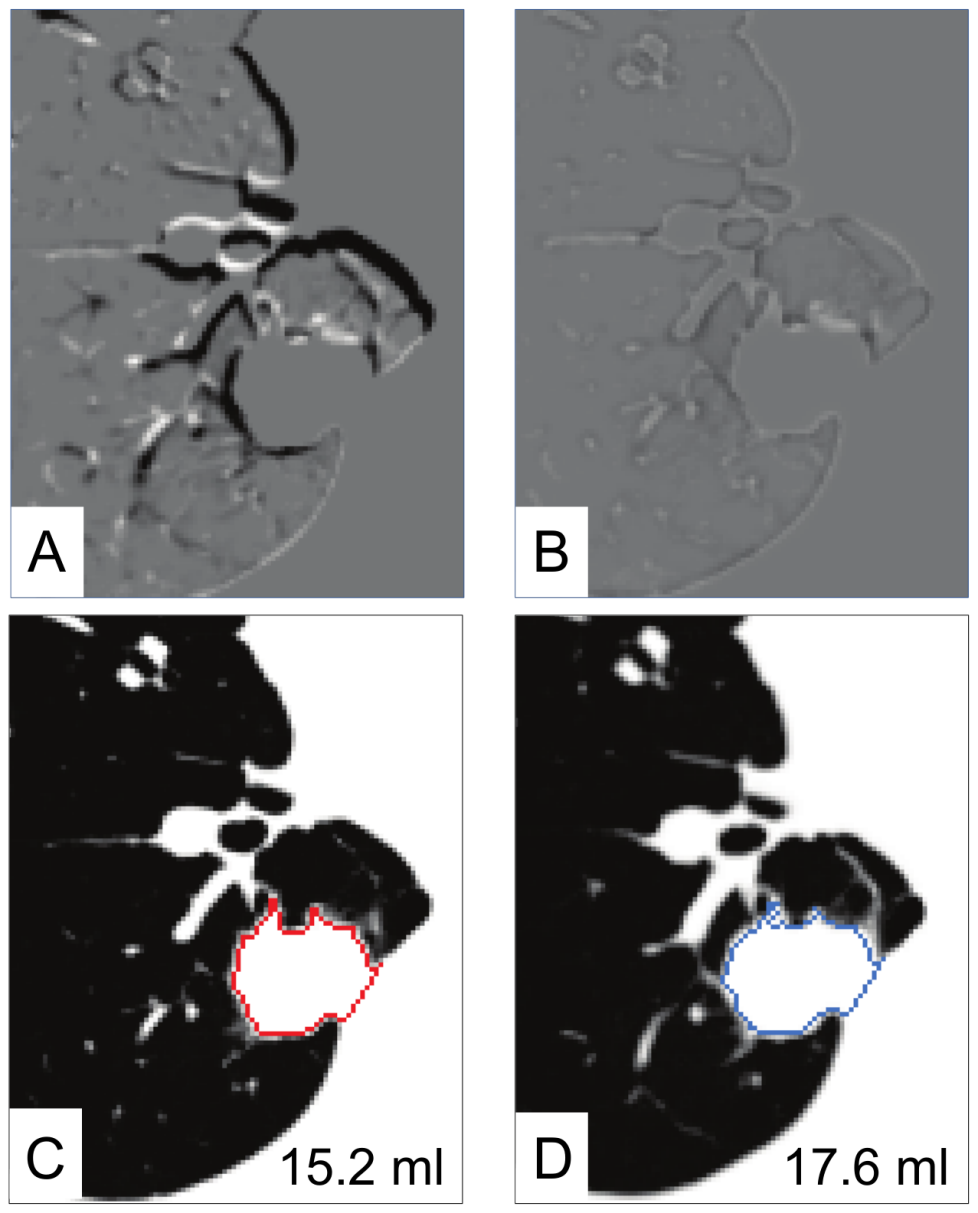
Automated and quantitative growth measurement using the cascading *α* LDDMM transformation matrix. Linear registration (A) shows some mis-registration as black or white linear structures, whereas LDDMM transformation (B) shows complete nodule registration, as well as the surrounding lung parenchyma. Once the nodule at the first time point is defined (C), the nodule definition can be automatically transformed to the second time point (E), which enables automated volume measurement.

The high quality registration of the cascading LDDMM ensures that most of the growth information is stored in the transformation matrix, which can be used to perform growth measurements. Fig. 5 demonstrates one of the volumetric approaches. In this demonstration, the subtraction image before LDDMM showed a dark ring around the nodule, which roughly suggested enlargement of the nodule in the follow-up image (Fig. 5A). After non-linear transformation with LDDMM, the nodules were completely registered (Fig. 5B). In Fig. 5C, the nodule was manually delineated at the first time point, which yielded the nodule volume of 15.2 ml. After perfect registration, the ROI of this nodule was transformed using the inverse transformation matrix, which automatically defined the nodule at the second time point (Fig. 5D). The volume of the growing nodule could be calculated as 17.6 ml (i.e., 15.8% volume increase).

### Comparison with B-spline Registration

**Figure 6:**
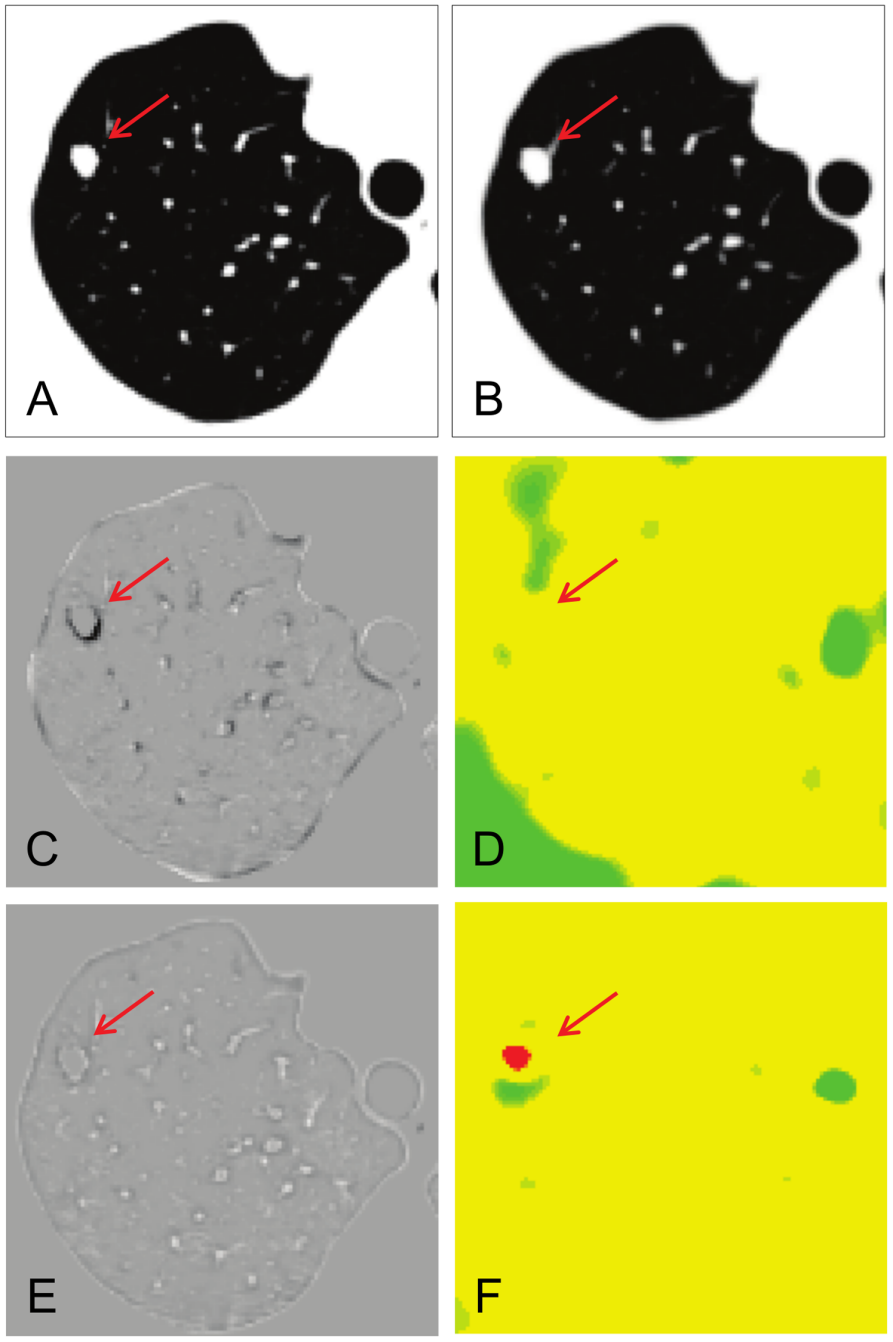
Comparison between cascading*α* LDDMM and B-spline registration. A metastatic nodule in the right upper lung (red arrow) was growing slightly at clinical follow-up (A: 1st time point, B: 2nd time point). C: Subtracted image with B-spline registration shows slight mis-registration along the lung parenchyma and a thin, rim-like difference around the nodule. D: Jacobian map obtained from B-spline registration is inhomogeneous and volume expansion is not clear. E: Cascading LDDMM shows complete registration. F: Jacobian map from cascading LDDMM obviously shows a red-colored spot, which corresponds to the growing nodule.

The landmark displacement of B-spline registration was sufficiently small, but significantly larger than that of cascading LDDMM. Fig. 6 shows that the metastatic nodule in the right upper lung was growing slightly at clinical follow-up. On the B-spline subtraction image (Fig. 6C), there were slight misalignments at the vessels and chest wall. The Jacobian map derived from B-spline registration (Fig. 6D) was inhomogeneous compared to that from cascading LDDMM (Fig. 6F). Cascading LDDMM showed complete registration at the metastatic nodules in serial CT (Fig. 6E). There was no rim-like difference on the subtraction image and a red-colored spot was clearly demonstrated on the color-coded Jacobian map with less artifacts (Fig. 6F). The result of B-spline registration showed a thin, rim-like difference around the nodule (Fig. 6C), although it was not distinct from other misalignments of the normal parenchyma. Volume expanding was not clearly detected on the Jacobian map (Fig. 6D) compared to cascading LDDMM.

## Discussion

### Necessity of Automated Analysis

A large number of patients undergoing screening for lung cancer have non-calcified nodules, and approximately half of the nodules are small, usually less than 5 mm in diameter, and most of them are benign [18]. On the other hand, primary lung cancer was discovered in 38% of patients who underwent video-assisted thoracic surgery for nodules of 1 cm or smaller detected on CT [19]. The median doubling time of lung cancer in volumetry before treatment was 181 days, with a very wide range [20], and serial comparisons of CT examinations within a few months up to years is quite important. In fact, size growth is the most important characteristic of malignancy except for tumors with predominantly ground-glass opacity [21]. Manual measurement of nodule growth on follow-up CT scans has been reported [20], [22], [23] and it is an accepted approach [24].

However, relatively high inter- and intra-observer variability was found in the measurement of lung tumor size on CT scans, which can lead to an incorrect interpretation of tumor response [25]. Accurate and objective size measurement and detection of newly emerged metastatic nodules are of similar importance for the evaluation of therapeutic responses in malignancy [26], [27]. In clinical evaluation, objective information additional to the original CT images is highly helpful. As in our study, temporal subtraction images and Jacobian maps derived from non-linear registration were useful, especially in patient follow-ups with multiple pulmonary metastases, because these patients often had numerous small metastatic nodules. Evaluation of therapeutic effect, such as appearing, disappearing, enlargement, or shrinkage of nodules is highly time-consuming work.

### Registration Accuracy by LDDMM

In this study, we applied LDDMM to the lung and found highly accurate registration results. The single LDDMM of low elasticity could serve most purposes, in which local severe deformation, such as tumor growth, are largely left untouched. Therefore, size differences are detected in the subtracted images. If the subtracted images are used qualitatively to assist image viewing, this level of accuracy is likely sufficient. The subtracted intensity (positive or negative values) could be color-coded and superimposed on the second time point images for such visual assistance.

The cascading LDDMM could remove most of the tissue deformation and the subtracted images approached null. Note that even the growing nodules were also transformed to the shape of the first time point, erasing the important growth information from the subtracted images. Indeed, if the transformation is perfect, the two images at different time points would become exactly the same, making the subtraction images virtually useless. Although this could be seen as a disadvantage of highly elastic transformation, the information about tissue transformation is stored in a transformation matrix, and the visualization of the transformation matrix, such as Jacobian maps, allows detection of growing or shrinking tumor. This method is also expected to detect the temporal change in ground-glass nodules, which are recommended to be followed by yearly CT for management [28], except when the lesion is so faint that it can barely be recognized, even on the original image. However, complete removal of the misalignment cannot always expected by the LDDMM, especially when there is a large amount of tumor growth, and, thus, both subtracted images and Jacobian maps have to be examined carefully (Fig. 4).

It should be noted that the diffeomorphic transformation cannot transform a newly emerged or vanished tumor, which violates the diffeomorphism and is invisible on Jacobian maps. Such structures, however, remain in the subtracted images. This difference may help to differentiate growing nodules that are detected in Jacobian maps and emerged nodules in the subtraction maps. Superimposition of the subtracted images (e.g., Figs. 2E and 3E) and color-coded Jacobian maps (e.g., Figs. 2F and 3F) could be a practical solution for comprehensive visualization of temporal change, and also help address the imperfect deformation accuracy.

We adopted a smooth reconstruction kernel for CT images, which were down-sampled before the registration process. Image noise was reduced in these steps, but the performance of registration tools would be influenced by the amount of noise in general. We have to be aware that our results are applicable for a given image resolution and SNR, and may not be applied to images with markedly different imaging parameters.

### Comparison to Former Automated CT Analysis Tools

The precision of *in vivo* volumetric analysis of nodules with an automatic volumetry software tool was sufficiently high in small pulmonary nodules, as well as in hepatic metastases [29], [30], [31]. A CAD system could successfully match 91% of all nodules detected in pairs of MDCT chest screening examinations, but it successfully assessed growth rates only in 55%, which was explained, in large part, by the CAD failure to detect and differentiate small nodules that have contact with adjacent anatomic structures [4], although this has recently been improved [32]. Registration is also largely affected by interval changes in size [5]. Segmentation enables better alignment and higher robustness [33], and has been used for tumor delineation and growth assessment [4], [34], [35]. However, it requires certain nodule models that may limit the capability of automated assessment. In this study, we applied a temporal subtraction method to detect the change in pulmonary nodules in serial CT images with a diffeomorphic transformation technology for registration of soft tissues. Our measurements (0.07±0.44 mm, on average) indicate that the registration accuracy approaches the image resolution for the cases used in this study, and the subtraction images and Jacobian maps could successfully detect small volume changes and metastasis sites.

### Comparison with Other Methods for Temporal Changes in Serial Lung CT Images

A number of registration methods for serial CT have been reported to aid radiologists in reading numerous slices of high-resolution CT images. Both linear and non-linear transformation methods have been used for image registration. Linear transformation, such as affine transformation [36], [37], costs less computational time and more robust against errors caused by over-fitting or trapping at a local minimum. However, the lung is highly deformable and the performance of linear registration may be limited for the coarse alignment of images to assist image comparison by radiologists. In our results with affine registration (Figs. 2C and 3C), numerous misalignments were found in the subtraction image. A more accurate, non-linear registration approach will be required if visualization of small temporal changes between serial CT images is desired.

On the other hand, non-linear transformation modifies the shape and volume of nodules and may remove information about the temporal changes in the subtracted images. Staring *et al.* [38], [39] and Zheng *et al.* [40] reported a non-linear registration method combined with a local rigid transformation to preserve the volume and shape of nodules. In this approach, the temporal changes were visualized on the subtraction images, but, the processing required additional steps, such as manual ROI placement around the tumor or segmentation before performing registration.

The basic concept of our study was to detect temporal changes fully automatically, using LDDMM, and to extract temporal change information from serial CT images through a combination of the subtraction image and the Jacobian map. Several previous reports have introduced a similar method in tumors and in demyelinating diseases of the brain [15], [41], [42]. For lung CT, the Jacobian map is often used to evaluate lung motion through breathing [43], [44]. To the best of our knowledge, the application of the combination of the Jacobian map and the subtraction image for the detection of temporal changes in lung nodules has not been reported. Cascading LDDMM clearly visualized the temporal changes in nodules on the Jacobian map as colored spots, and there was no need to perform a manual step, such as ROI definition. The registration process without any manual step would facilitate the use of automatic processing before reading CT images.

To evaluate the temporal changes in lesions using the Jacobian map properly, the deformation for image registration should be biologically plausible. LDDMM provides an elegant approach for this challenging task; it maintains topology throughout the iteration–connected structures remain connected and separated structures remain separated. Other non-linear methods, such as B-spline (polynomial functions) [10], [11] and optical flow [45], [46] can register corresponding grid points, but have the potential to violate topology, although they have been improved by incorporating regularization steps to avoid these issues [47]. The result of B-spline registration was excellent in our comparative trial, although landmark displacement was significantly larger than cascading LDDMM. Furthermore, to visualize small changes, the Jacobian map should reflect pixel-wise volume change. In our result, the volume change of small nodules was not clearly visualized on the Jacobian map obtained from B-spline registration (Fig. 6D), while that of the cascading LDDMM could visualize well the volume change (Fig. 6F).

### Possible Applications to Other Regions

In an analysis of recurrent colorectal cancer, small pulmonary nodules and liver metastases were slightly better detected on CT images by registration with PET images, whereas metastases to the peritoneum, lymph nodes, and bone were more easily detected on PET only [48]. However, without using other modalities, the demonstration of volume changes in these areas by registering serial CT images will facilitate the detection of malignancy. Aside from malignancy, there are many other situations where volume change in serial observation is important. LDDMM analysis of serial CT image pairs may be applied to benign changes, such as regression of interstitial pneumonia [49] and body fat reduction of obese patents [50].

### Limitations

The LDDMM-based approach is not perfect. Even with the cascading LDDMM, some small structures in the lung may still be misaligned, as shown in Fig. 2. If inspiratory volume is quite different between the first and second time point, registration error may ensue. The choice of the ratio was empirically set in this study and further investigation in a future study is necessary to define optimized parameters (e.g., the range and step of values). However, we can identify these misalignments of the normal parenchyma as a typical presentation of striated shadows on a subtraction image and recognize these as vessel misalignment. However, if there is drastic change in lung shapes between two time points, such as a substantial size change in lung nodules or a density change in the lung field (e.g., pneumonia, congestion, etc.), transformation would fail at some point. Particularly when these drastic changes occurred adjacent to the chest wall or cardiac outline, they were distorted and extended into the lung field. Therefore, quantitative criteria to measure the success of transformation, such as residuals of the cost function values after transformation, would be an important target of future investigation. If registration of nodule shape and size is not perfect, the evaluation of the temporal volume change of a nodule, calculated from the transformation matrix, would also become inaccurate, although the accurate quantification of tumor growth would be less important if there is a drastic increase in tumor size. Even when complete registration is achieved, as shown in Figure 4, the appearance and disappearance or shrinkage and enlargement of a nodule can be expressed in certain patterns that are relatively easy to comprehend. However, it might be complicated if two additional images have to be compared with original images. Improving the fashion of visualization will be an issue in the future. One obvious solution is to create a hybrid image by combining a gray-scale subtracted image and a color-coded Jacobian map. Creating another new image by automatically interpreting the subtraction and Jacobian map beyond a simple overlay, or three-dimensional mapping, like maximum intensity projection (MIP), might be beneficial choices.

Another limitation resides in the requirement for computational resources. In addition, this method requires a cluster computer with a large amount of RAM (more than 32 GB to analyze one data), and is currently not feasible on a desktop PC. The code used in this study was not parallelized, and we believe there is ample room for faster calculation, which will be an important future effort.

In conclusion, LDDMM provided highly accurate registration in serial chest CT images, and the temporal subtraction images with Jacobian maps can help radiologists to determine the changes in pulmonary nodules.

## Supporting Information

Text S1 (DOCX)
