# Supplementary material for: Detection of Time-Varying Structures by Large Deformation Diffeomorphic Metric Mapping to Aid Reading of High-Resolution CT Images of the Lung
Source: PLoS One. 2014 Jan 13;9(1):e85580. doi: 10.1371/journal.pone.0085580 (PMC3890326; doi:10.1371/journal.pone.0085580)
Supplement: Text S1 — (DOCX) [file pone.0085580.s001.docx]

**Text S1. LDDMM-based non-linear registration.**

The linearly normalized images were then further transformed for more precise image-matching using LDDMM. The LDDMM was performed according to a previous publication. Briefly, the LDDMM algorithm computes a transformation, $\phi:\Omega\to\Omega$, where $\Omega\subseteq R^{3}$ is the 3D cube on which the data are defined, and the observed anatomical image $I_{1}$ and template image $I_{0}$ are given and registered such that $I_{1}=I_{0}\circ\phi^{-1}$. The computed transformation, $\phi$, is the end point, $\phi=\varphi_{1}$, of a flow of velocity fields, $\nu_{t}\in V,t\in\left[0,1\right]$, given by the ordinary differential equation, where $\varphi_{0}$ is the identity transformation, $\varphi_{0}\left(x\right)=x,\forall x\in\Omega$. Then, the optimal transformation is calculated by integrating the vector field that is found by minimizing the following equation.

$\nu^{\wedge} =\mathop{\mathrm{argmin}}\limits_{\nu:\frac{d\varphi}{dt}=\nu_{t}\left(\varphi_{t}\right)} \left(\int_{0}^{1} \left\|\nu_{t}\right\|_{V}^{2}dt+\frac{1}{\sigma^{2}}\left\|I_{0}\circ\phi^{-1}-I_{1}\right\|_{L^{2}}^{2}\right)$ , (1)

As shown by Dupuis [[1](#_ENREF_1)], enforcing a sufficient amount of smoothness on the elements in the space of allowable vector fields, $V$, ensures that the solution to the differential equation, is in the space of diffeomorphism. Smoothness is enforced throughout by defining the norm on the space, $V$, of smooth velocity vector fields through a differential operator, $L$, which generally represents Laplacian powers such that $\left\|f\right\|_{V}^{2}=\left\|Lf\right\|_{L^{2}}^{2}$, where is the standard $L^{2}$ norm for square integrable functions defined on $\Omega$. The differential operator, $L$, is defined as $L=-\alpha\nabla^{2}+\gamma I$, where $I$ is the identity operator and is the Laplacian operator. The gradient of the cost in Eq. (1) is:

$\nabla_{\nu}E_{t}=2\nu_{t}-K\left\{\frac{2}{\sigma^{2}}\left|D\varphi_{t,1}\right|\nabla J_{t}^{0}\left(J_{t}^{0}-J_{t}^{1}\right)\right\}$ , (2)

where the notation $\varphi_{s,t}=\varphi_{s}\circ\varphi_{t}^{-1}$ is used. In Eq. (2), $J_{t}^{0}=I_{0}\circ\varphi_{t,0}$ and $J_{t}^{1}=I_{1}\circ\varphi_{t,1}$. $\left|Df\right|$ is the determinant of the Jacobian matrix. $K:L_{2}\left(\Omega,R^{3}\right)\to V$ is a compact self-adjoint operator, defined by $\left\langle a,b\right\rangle_{L^{2}}=\left\langle Ka,b\right\rangle_{V}$, which satisfies $K\left(L^{\text{†}}L\right)g=g$ for any smooth vector field $g\in V$ . The parameter $\sigma$ provides weighting between data-matching and smoothness regularization terms. In the LDDMM algorithm, Eq. (1) is solved with a gradient descent algorithm using Eq. (2). In Eq. (2), the effect of the operator, $K\left(L^{\text{†}}L\right)^{-1}$, is low-pass filtering. The parameters $\alpha$ and $\gamma$ define the magnitude and frequency response of this filter.

**Reference**

1. Paul Dupuis UG, Michael I. Miller (1998) Variational problems on ﬂows of diffeomorphisms for image matching. Quarterly of Applied Mathematics 56: 587–600.
